# The Role of Oral Nutritional Supplements in Head and Neck Cancer Patients Undergoing Chemoradiotherapy

**DOI:** 10.3390/healthcare12202070

**Published:** 2024-10-17

**Authors:** Mohamed Abouegylah, Suchira Subodini Udugamasooriya, Ahmed Adel Ahmed, Kerem Tuna Tas, Philipp Lishewski, Edgar Smalec, Gertrud Schmich, Hilke Vorwerk, Fabian Eberle, Sebastian Adeberg, Ahmed Gawish, Abdelsalam A Ismail

**Affiliations:** 1 Department of Clinical Oncology, Faculty of Medicine, Alexandria University, Alexandria 21563, Egypt; suchirasu@gmail.com (S.S.U.); salam61@yahoo.com (A.A.I.); 2 Department of Clinical Oncology, Ayadi Al-Motakbal Oncology Hospital, Alexandria 21563, Egypt; a7med_adel99@yahoo.com; 3 Marburg Ion-Beam Therapy Center (MIT), Department of Radiotherapy and Radiation Oncology, Marburg University Hospital, 35039 Marburg, Germany; keremtuna.tas@uk-gm.de (K.T.T.); philipp.lishewski@uk-gm.de (P.L.); edgar.smalec@uk-gm.de (E.S.); gertrud.schmich@uk-gm.de (G.S.); hilke.vorwerk@uni-marburg.de (H.V.); fabian.eberle@uk-gm.de (F.E.); sebastian.adeberg@uk-gm.de (S.A.); 4 Department of Radiotherapy and Radiation Oncology, Marburg University Hospital, 35039 Marburg, Germany; 5 Department of Radiotherapy and Radiation Oncology, Philips University, 35039 Marburg, Germany

**Keywords:** head and neck cancer, oral nutritional supplementation (ONS), radiotherapy, chemotherapy, radiation toxicities

## Abstract

Purpose: This study aimed to assess the impact of oral nutritional supplements (ONS) on nutritional intake, body weight, and body composition in head and neck cancer (HNC) patients undergoing chemoradiotherapy. The study evaluated whether ONS could prevent treatment-related nutritional deterioration. Methods: This prospective observational pilot study included 30 HNC patients randomized into two groups: ONS (n = 15) and No ONS (n = 15). All participants underwent chemoradiotherapy, with the ONS group receiving 200 mL of a high-calorie, high-protein supplement twice daily. Nutritional status, including body weight, BMI, fat mass, fat-free mass, and bone mass, was assessed at three time points: baseline, mid-treatment, and end of treatment. Data were analyzed using the Mann–Whitney U test, with a *p*-value of ≤0.05 considered statistically significant. Results: At baseline, there were no significant differences between the two groups in body weight, BMI, or body composition. By the end of radiotherapy, the No ONS group showed significant reductions in body weight (*p* < 0.001), BMI (*p* < 0.001), fat mass (*p* < 0.001), and fat-free mass (*p* < 0.001), while the ONS group maintained more stable nutritional parameters. Acute radiotherapy toxicities, including nausea, dysphagia, and oral mucositis, were not significantly different between the two groups. Conclusion: ONS effectively mitigates weight loss and preserves body composition in HNC patients undergoing chemoradiotherapy. While no significant reduction in radiation-induced toxicities was observed, the nutritional benefits of ONS support its use in preventing malnutrition in this patient population. Larger studies are needed to further validate these findings.

## 1. Introduction:

Head and neck cancer (HNC) is recognized as the seventh most common type of malignancy worldwide [1]. Treatment for HNC, particularly chemoradiotherapy (CRT), is linked with several side effects, including oral mucositis, xerostomia (dry mouth), and dysgeusia (altered taste), all of which can significantly affect a patient’s ability to maintain adequate nutritional intake [2,3]. Additionally, the presence of the tumor itself may lead to mechanical issues, such as difficulties in chewing and swallowing [4], further complicating the act of eating and potentially making it painful or uncomfortable for the patient [5].

The symptoms associated with head and neck cancer (HNC) can lead to reduced food consumption, which is directly linked to weight loss, malnutrition, diminished quality of life, increased infection risk, higher rates of hospital readmissions, prolonged hospital stays, and increased mortality [6,7,8]. During treatment, changes in the regularity of food consumption among HNC patients have been observed, potentially disrupting their energy balance and decreasing their intake of macronutrients [9]. Additionally, these patients often exhibit deficiencies in micronutrients, including vitamins D, E, and C, as well as folate, calcium, iron, and magnesium, necessitating oral nutritional supplementation (ONS) to meet recommended levels [10,11]. Micronutrients are essential for enzymatic reactions critical to overall metabolism [12].

The United Kingdom National Multidisciplinary Guidelines highlight the importance of dietary support as a core component of HNC treatment. It is advised that nutritional interventions be implemented during therapy to prevent weight loss, improve meal consumption, and minimize treatment interruptions [13]. In cases of inadequate oral intake, the use of oral nutritional supplements (ONS) and tube feeding is recommended. Studies have shown that HNC patients who receive nutritional counseling and support experience improvements in weight loss, quality of life, and survival [14,15].

Thus, providing comprehensive nutritional support to HNC patients, including the use of oral nutritional supplements, is imperative. There is currently a gap in research regarding the precise amounts of nutrients obtained from diet and supplements during treatment. The literature explores the effects of individual nutritional counseling and the use or non-use of ONS on outcomes, such as weight, quality of life, mortality, and nutritional status. However, these factors are abstract, and more studies are needed that focus on quantifying nutrient intake, a component often neglected in clinical practice and under-researched due to its complex analysis. This study aims to fill this gap by evaluating both macro- and micronutrient intake during treatment, particularly in the short term. This approach is crucial for enabling regular monitoring and addressing potential cumulative nutritional deficits that often go underestimated in these studies.

Moreover, there is limited information regarding the frequency of insufficient food intake among HNC patients. Identifying specific points during treatment when dietary intake is most affected is vital. Such timely assessments of nutritional changes can help mitigate adverse effects through nutritional counseling. Furthermore, by not only providing nutritional guidance but also identifying specific needs, it is possible to prevent weight loss and ensure that patients who may not display overt signs of malnutrition are not overlooked.

Our hypothesis is that both macro- and micronutrient intake are reduced in HNC patients during their treatment. This study aims to quantify the frequency of insufficient energy and nutrient intake and analyze changes in body weight composition (BWC) in patients undergoing chemoradiation.

## 2. Materials and Methods

### 2.1. Study Design and Setting

This prospective observational pilot study was conducted among head and neck cancer (HNC) patients undergoing chemoradiotherapy. Recruitment occurred at the Clinical Oncology Department of a tertiary university hospital, which serves as the regional referral center for HNC patients receiving antineoplastic treatment.

### 2.2. Participants

A total of 30 patients with histopathologically confirmed head and neck cancer (stages 1 to 4) were enrolled. Patients were eligible if they were at least 18 years of age, had no prior anti-cancer treatments, and demonstrated an ECOG performance status of 0 or 1. Exclusion criteria included inability to manage oral feeding, requirement for inpatient care, BMI over 30, medical conditions precluding a high-protein diet (such as liver or renal insufficiency), or pre-existing malnutrition.

### 2.3. Study Groups

Participants were randomly assigned to one of two groups using systematic random sampling. The Oral Nutritional Supplements (ONS) group (study arm) consisted of 15 patients, and the No ONS group (control arm) also consisted of 15 patients. All participants underwent radiotherapy, with or without concurrent chemotherapy.

### 2.4. Nutritional Procedure

Patients in the ONS group received a twice-daily dose of 200 mL of Medidrink Onco. Each 100 mL of ONS had the following nutritional composition:

1. Energy: 920 KJ
2. Fat: 14.2 g
3. Carbohydrate: 11 g
4. Protein: 11 g
5. Water: 70 mL
6. Fiber: 2 g
7. Minerals: Sodium, Potassium, Calcium, Magnesium, Phosphorus, and Chloride.
8. Trace Elements: Iron, Zinc, Copper, Manganese, Iodine, Fluoride, Chromium, Molybdenum, and Selenium.
9. Vitamins: Vitamin A, Thiamin, Niacin, Vitamin B6, Riboflavin, Vitamin B12, Pantothenic acid, Biotin, Folic acid, Vitamin C, Vitamin D, Vitamin E, and Vitamin K.

Supplements were provided on a weekly basis.

### 2.5. Data Collection

Data was collected at baseline, mid-way, and at the end of radiotherapy. Demographic and cancer-related data, including tumor location, histology, grade, and stage, were obtained from medical records. Patients’ weight, BMI, and body composition were assessed at these intervals. Acute toxicity was evaluated using established grading systems.

### 2.6. Body Composition Assessment

Body composition was assessed using a device incorporating eight leads: four hand electrodes and four foot electrodes. The device used two predetermined frequencies to yield precise body composition data. Patient information, such as gender, year of birth, and height, was input into the device. Patients were instructed to stand on the device while holding the hand pieces, and the body composition data were displayed on the screen.

### 2.7. Statistical Analysis

Data were analyzed using SPSS version 22. Numerical data were summarized as median and range. The Mann–Whitney U test was used to determine statistical differences between the ONS and control groups. A *p*-value of ≤0.05 was considered statistically significant.

### 2.8. Ethical Considerations

All patients provided informed consent before participation. The study protocol was approved by the institutional ethics committee, and all procedures were conducted in accordance with the ethical standards of the responsible committee on human experimentation and with the Helsinki Declaration of 1975, as revised in 2000.

## 3. Results

### 3.1. Patient Characteristics

The demographic characteristics of the patients examined in this study, which included factors such as age, gender, height, and smoking patterns, did not show any substantial variations or statistically significant disparities between the two groups, as shown in Table 1.

### 3.2. Tumor Characteristics

Analysis of tumor characteristics, including tumor site, stage, histology, and differentiation, revealed no significant discrepancies between the groups that received ONS intervention and those that did not. However, a notable majority of patients in this study had larynx cancer, accounting for 73.33% in the ONS group and 60% in the No ONS group. The primary histology type identified was Squamous Cell Carcinoma, constituting 80% in the ONS group and 93.33% in the No ONS group. The distribution of tumor site and histology type varied somewhat between the groups, as represented in Table 2.

### 3.3. Radiotherapy Regime

This study examined various radiotherapy regimens, including definitive RT, postoperative RT with or without chemotherapy, and induction chemotherapy followed by RT. No statistically significant difference was observed between the groups regarding the RT regimen. However, a significant difference was found in the total radiotherapy dose (*p* = 0.002). In the ONS group, 80% of the patients received RT alone, with 42.86% receiving a dose of 60 Gy. In contrast, 73.33% of the No ONS group received concurrent chemoradiotherapy, with 64.29% receiving a dose of 70 Gy. These findings indicate variability in the choice of radiotherapy dose and regimen among the groups (Table 3).

### 3.4. Comparison of Nutritional Status

Patients in this study were assessed for various nutritional parameters at different time points during their radiotherapy (RT) course. The first assessment took place at the beginning of the RT course, corresponding to the first week of treatment. At this time, there were no statistically significant differences between the two groups (ONS and No ONS) in terms of median body weight (77.8 kg vs. 75.70 kg; *p* = 0.373), BMI (28.2 kg/m^2^ vs. 28.15 kg/m^2^; *p* = 0.254), fat mass (24.5 kg vs. 22.7 kg; *p* = 0.071), fat-free mass (51.55 kg vs. 55.5 kg; *p* = 0.556), and bone mass (3.1 kg vs. 3.0 kg; *p* = 0.188). Therefore, the baseline nutritional status was similar in both groups.

The second assessment was conducted in the middle of the RT course, which corresponded to the third or fourth weeks into treatment. Again, no statistically significant differences were found between the two groups in terms of median body weight (76.1 kg vs. 74.85 kg; *p* = 0.421), BMI (28.20 kg/m^2^ vs. 27.37 kg/m^2^; *p* = 0.215), fat mass (23.45 kg vs. 20.40 kg; *p* = 0.168), fat-free mass (53.10 kg vs. 54.00 kg; *p* = 0.727), and bone mass (3.10 kg vs. 2.95 kg; *p* = 0.310). Therefore, the middle of the RT course nutritional status was comparable between the ONS and No ONS groups. Moreover, the percentage change or pairwise comparisons of the nutritional assessing parameters from baseline to the middle of the RT course did not show any statistically significant differences.

The final assessment was conducted at the end of the RT course, which corresponded to the sixth or seventh weeks into treatment. Similar to the previous assessments, there were no statistically significant differences between the two groups in terms of median body weight (73.90 kg vs. 72.95 kg; *p* = 0.383), BMI (27.64 kg/m^2^ vs. 26.34 kg/m^2^; *p* = 0.054), fat mass (23.45 kg vs. 19.30 kg; *p* = 0.073), fat-free mass (52.50 kg vs. 53.60 kg; *p* = 0.872), and bone mass (3.00 kg vs. 2.95 kg; *p* = 0.310). Thus, the nutritional status at the end of the RT course was not significantly different between the ONS and No ONS groups. However, when considering the changes over time, the No ONS group showed statistically significant reductions in body weight (*p* < 0.001), BMI (*p* < 0.001), fat mass (*p* < 0.001), fat-free mass (*p* < 0.001), and bone mass (*p* = 0.031) throughout the treatment period. On the other hand, the ONS group did not show statistically significant reductions in these nutritional values, despite some reductions being observed.

Furthermore, pairwise comparisons within the No ONS group revealed that the loss of nutritional values was particularly evident towards the end of the RT course. The *p* values for the mid-end of RT vs. the beginning-end of RT comparisons were significant for body weight (0.007 and <0.001), BMI (0.007 and <0.001), fat mass (0.004 and 0.001), and fat-free mass (0.032 and <0.001), indicating a statistically significant deterioration in nutritional status after the middle of the RT course. However, there was no significant reduction in bone mass in the pairwise comparison. Therefore, the results suggest that while the ONS group did not show statistically significant reductions in nutritional values, the No ONS group experienced significant deterioration in nutritional status, especially towards the end of the RT course.

**Figure 1:**
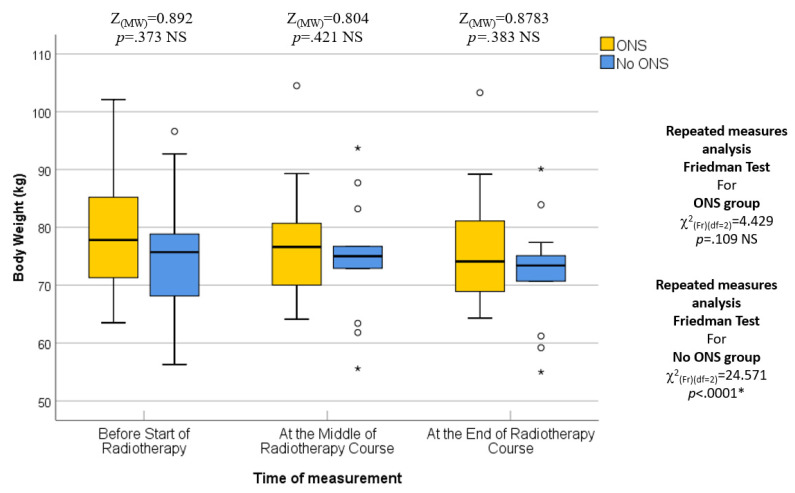
Box and whisker graph of body weight (kg) in the studied groups: The thick line in the middle of the box represents the median, the box represents the inter-quartile range (from 25th to 75th percentiles), and the whiskers represent the minimum and maximum, excluding outliers (circles) and extremes (asterisks). * statistically significant.

**Figure 2:**
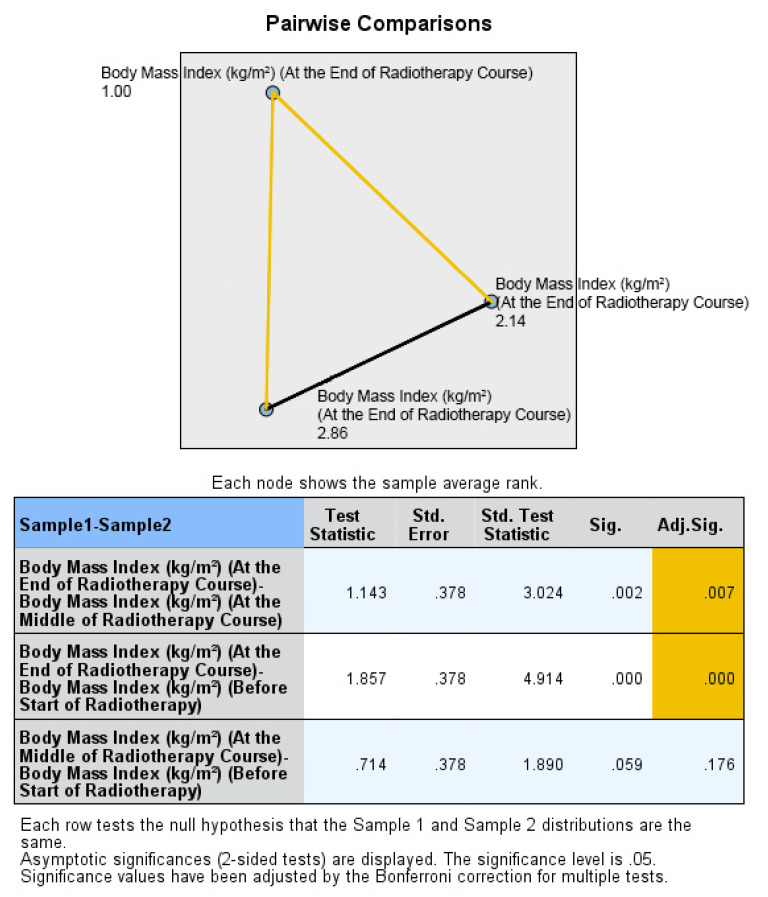
Box and whisker graph of body weight percentage change (%) in the studied groups: The thick line in the middle of the box represents the median, the box represents the inter-quartile range (from 25th to 75th percentiles), and the whiskers represent the minimum and maximum, excluding outliers (circles).

**Figure 3:**
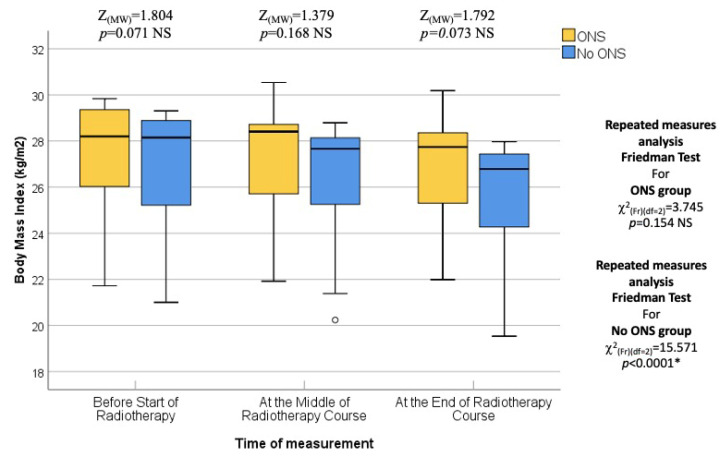
Box and whisker graph of body mass index (kg/m^2^) in the studied groups: The thick line in the middle of the box represents the median, the box represents the inter-quartile range (from 25th to 75th percentiles), and the whiskers represent the minimum and maximum, excluding outliers (circles). * statistically significant.

**Figure 4:**
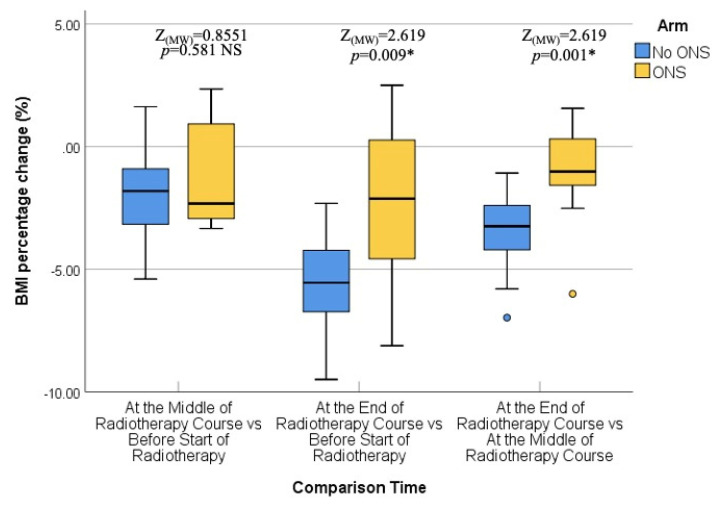
Box and whisker graph of body mass index percentage change (%) in the studied groups: The thick line in the middle of the box represents the median, the box represents the inter-quartile range (from 25th to 75th percentiles), and the whiskers represent the minimum and maximum, excluding outliers (circles). * statistically significant.

**Figure 5:**
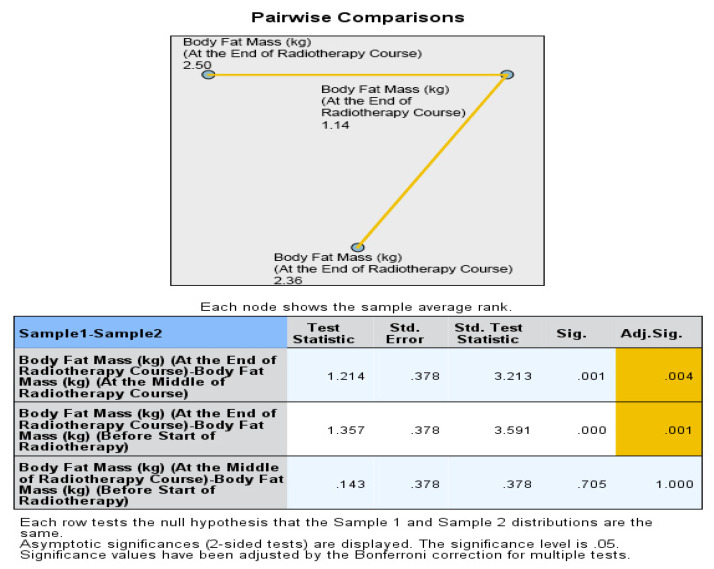
Box and whisker graph of body fat mass (kg) in the studied groups: The thick line in the middle of the box represents the median, the box represents the inter-quartile range (from 25th to 75th percentiles), and the whiskers represent the minimum and maximum, excluding outliers (circles).

**Figure 6:**
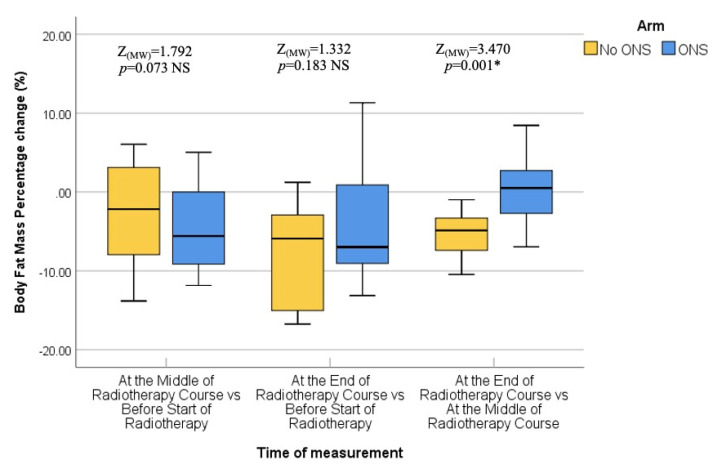
Box and whisker graph of body fat mass percentage change (%) in the studied groups: The thick line in the middle of the box represents the median, the box represents the inter-quartile range (from 25th to 75th percentiles), and the whiskers represent the minimum and maximum. * statistically significant.

**Figure 7:**
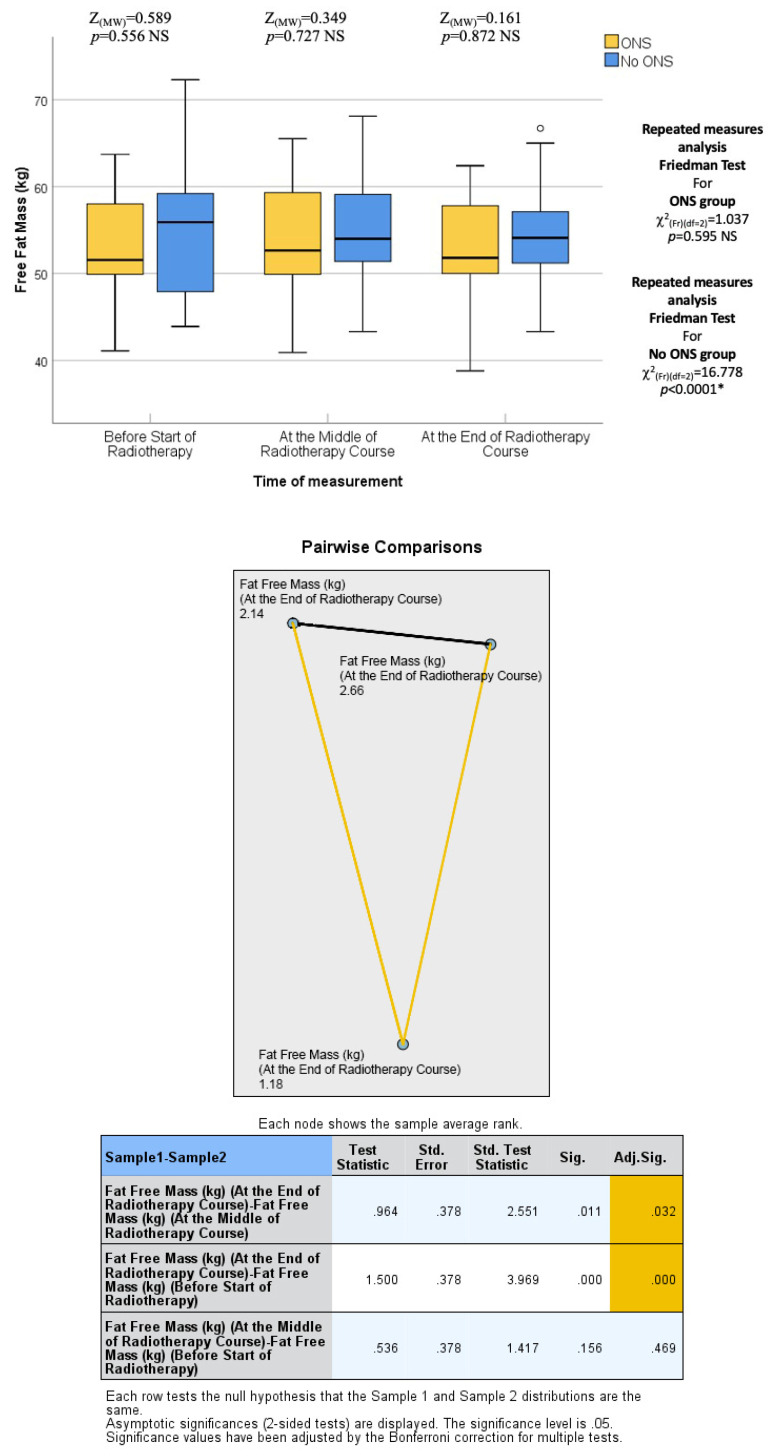
Box and whisker graph of fat-free mass (kg) in the studied groups: The thick line in the middle of the box represents the median, the box represents the inter-quartile range (from 25th to 75th percentiles), and the whiskers represent the minimum and maximum after excluding outliers (circles). * statistically significant.

**Figure 8:**
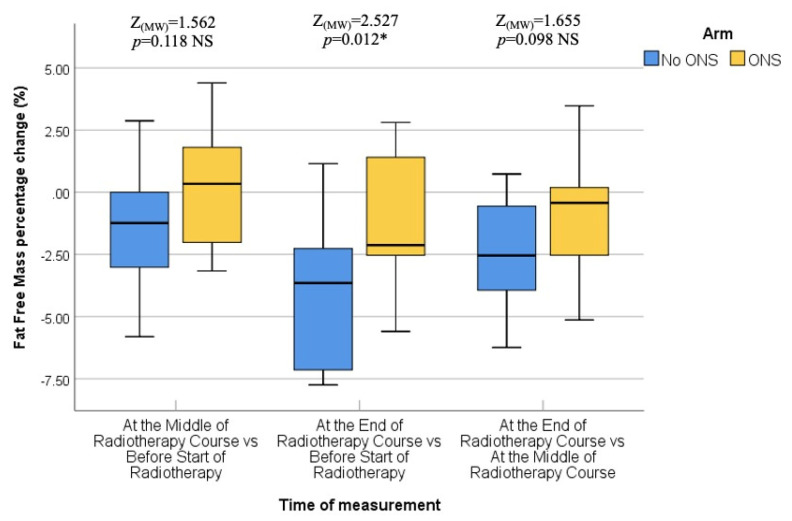
Box and whisker graph of fat-free mass (%) in the studied groups: The thick line in the middle of the box represents the median, the box represents the inter-quartile range (from 25th to 75th percentiles), and the whiskers represent the minimum and maximum. * statistically significant.

**Figure 9:**
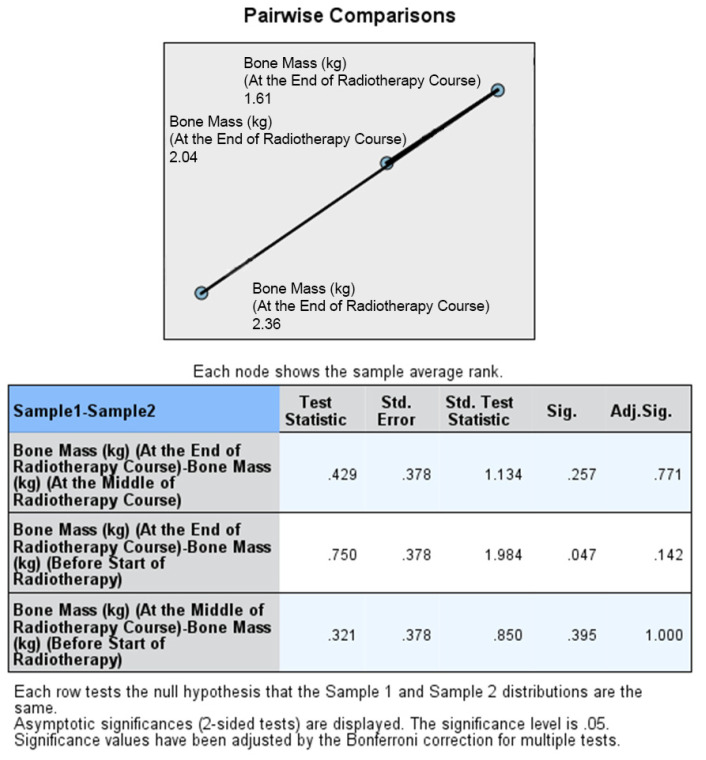
Box and whisker graph of bone mass (kg) in the studied groups: The thick line in the middle of the box represents the median, the box represents the inter-quartile range (from 25th to 75th percentiles), and the whiskers represent the minimum and maximum after excluding outliers (circles).

**Figure 10:**
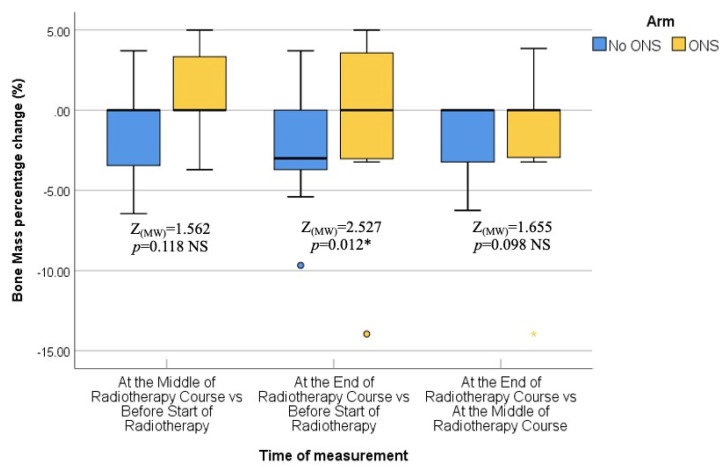
Box and whisker graph of bone mass (%) in the studied groups: The thick line in the middle of the box represents the median, the box represents the inter-quartile range (from 25th to 75th percentiles), and the whiskers represent the minimum and maximum. * statistically significant.

In addition, the percentage alteration of nutritional parameters also exhibited a statistically significant decrease in the group that did not receive oral nutritional supplements (ONS) when compared to the group that did receive ONS after the middle of the radiation therapy (RT) course. The *p*-values for the changes in body weight percentage and BMI percentage between the middle and baseline, end and middle, and end and baseline of the RT course were found to be 0.851, 0.001, and 0.009, respectively. The percentage change in fat mass demonstrated a significant alteration between the end and middle of the RT course (*p* = 0.001), while the percentage change in fat-free mass was found to be significant between the end and baseline (*p* = 0.012). On the other hand, there was no statistically significant alteration observed in the percentage change of bone mass. All the data are presented in the form of Tables (Table 4, Table 5, Table 6, Table 7 and Table 8) as well as in various figures (Figure 1, Figure 2, Figure 3, Figure 4, Figure 5, Figure 6, Figure 7, Figure 8, Figure 9 and Figure 10).

### 3.5. Comparison of Radiotherapy Side Effects

In the current investigation, we conducted a comparison of nausea, dysphagia, oral mucositis, xerostomia, and dermatitis in order to assess the acute radiation toxicity. The incidence of nausea was observed in 21.43% (n = 3/14) of the participants in the ONS group and 7.14% (n = 1/14) in the No ONS group; the *p*-value was determined to be 0.589. Conversely, dysphagia was present in 85.71% (n = 12/14) of the participants in the ONS group and 78.57% (n = 11/14) in the No ONS group, with a *p*-value of 1.00. The comparison between the two groups did not yield any statistically significant findings. It is important to note that the majority of participants in both groups experienced Grade 1 oral mucositis, with proportions of 78.57% in the ONS group and 71.43% in the No ONS group, and this discrepancy was not statistically significant (*p* = 0.396). Likewise, the presence of xerostomia and dermatitis did not exhibit any statistically significant disparities between the two groups, with *p*-values of 0.256 and 0.463, respectively, as shown in Table 9.

## 4. Discussion

The present study demonstrates that patients who did not receive oral nutritional supplementation (ONS) experienced a significant decline in their nutritional status during radiotherapy (RT). Patients compliant with ONS did not show significant weight loss, while non-compliant patients exhibited notable reductions in body weight, BMI, fat mass, and fat-free mass. These findings align with previous research by Hopanci Bicakli et al. [16], which indicated that body mass index, weight, fat percentage, fat mass, fat-free mass, and muscle mass did not decrease significantly in compliant patients, whereas these indices decreased significantly in non-compliant patients from baseline to the end of treatment (*p* < 0.001). Similarly, a Malaysian study [17] found that non-compliant patients had higher percentages of weight loss and significant reductions in body weight and muscle mass at the end of RT. Another study by Alhambra et al. [18] highlighted that early nutritional support therapy before the start of RT resulted in a lower BMI and significant loss of fat-free mass at the end of treatment.

In comparing acute radiation toxicities, including nausea, dysphagia, oral mucositis, xerostomia, and dermatitis, this study found no statistically significant differences between the two groups. However, Hopanci Bicakli et al. [16] reported a higher incidence of severe mucositis in compliant patients, and Imai et al. [19] noted that ONS use resulted in a lower incidence of grade 2 or higher dermatitis in HNC patients. Despite these differences, the current study did not find significant differences in the occurrence of dermatitis between the groups.

Although a higher percentage of patients in the ONS group tolerated food better than those in the No ONS group, this difference was not statistically significant. Previous research [2,3] has shown a decrease in the consumption of normal and soft diets during RT, with an increase in liquid diets and tube feeding. Another study [20] indicated a decrease in oral intake before RT, underscoring the importance of ONS during treatment. Most patients in this study received ONS for more than 4 weeks, suggesting its tolerability and potential benefits.

This study evaluated patients’ adherence to treatment by examining their completion of scheduled treatments. No significant difference was found in treatment discontinuation due to toxicity between the groups. However, a Chinese study [21] on locally advanced nasopharyngeal carcinoma reported a significantly higher rate of suspension or delay of RT in the control group compared to those receiving prophylactic ONS.

This prospective randomized study provided real-time data and utilized Bioelectrical Impedance Analysis (BIA) as a non-invasive, cost-effective method to evaluate patients’ nutritional status. BIA allowed for timely monitoring, which was crucial given the financial and time constraints of the trial. An important outcome was that a subset of HNC patients received necessary nutritional supplementation during their treatment.

Several limitations of this study should be acknowledged. The relatively small sample size limits the generalizability of the findings, necessitating caution when interpreting the results. The absence of a qualified nutrition professional’s involvement is another limitation, as their presence would have enhanced the study’s validity and reliability through accurate quantification of nutritional intake and provision of comprehensive nutritional counseling.

Additionally, the study excluded underweight and obese patients, leaving the efficacy of ONS in these populations unexplored. This exclusion limits the generalizability of the findings to a broader patient population.

Further research is needed to confirm and validate these results. Future studies should include larger sample sizes encompassing all HNC patients to determine the applicability of these findings to a wider patient population. Replication of this study with a larger sample size will help establish the robustness and reliability of the results.

## 5. Conclusions

This study demonstrates that oral nutritional supplementation (ONS) plays a vital role in maintaining nutritional status in head and neck cancer (HNC) patients undergoing chemoradiotherapy. Patients who received ONS experienced significantly less weight loss and better preservation of body composition compared to those who did not receive supplementation. These results underscore the importance of integrating ONS into the nutritional management of HNC patients to mitigate treatment-related malnutrition and preserve lean body mass. While no significant differences in radiation-induced toxicities were observed, the findings suggest that ONS can effectively counteract the nutritional decline typically seen during treatment. Given the study’s limitations, including a small sample size and the exclusion of underweight and obese patients, further research with a larger cohort is warranted to confirm the benefits of ONS and to explore its impact on diverse patient populations. Expanding research in this area will be crucial to developing comprehensive nutritional guidelines for HNC patients undergoing chemoradiotherapy.

## Figures and Tables

**Table 1:** Demographic criteria of head and neck cancer patients.

Demographic Criteria		Groups				*p* Value
		ONS (n = 15)		No ONS (n = 15)		
Age (years)		60.00 (29.00–79.00)		55.00 (20.00–68.00)		*p* = 0.253
Sex distribution		Male	Female	Male	Female	*p* = 1.000
Male (n = 23) (76.67%)		11	4	12	3	
Female (n = 7) (23.33%)		73.33%	26.67%	80.00%	20.00%	
Height (cm)		170.00 (162.00–185.00)		166.00 (152.00–183.00)		*p* = 0.546
Smoking						*p* = 1.000
NO	YES	NO	YES	NO	YES	
10 (40.00%)	20 (60.00%)	5 (30.33%)	10 (66.67%)	5 (30.33%)	10 (66.67%)	

**Table 2:** Tumor characteristics of head and neck cancer patients.

Tumor Characteristics		Groups				*p* Value
		ONS (n = 15)		No ONS (n = 15)		
Tumor site						*p* = 0.591
Larynx (n = 20) (66.67%)		11 (73.33%)		9 (60.00%)		
Hypopharynx (n = 2) (6.67%)		0 (00.00%)		2 (13.33%)		
Oral cavity (n = 5) (16.67%)		2 (13.33%)		3 (20.00%)		
Nasal cavity and para nasal sinus (n = 3) (10.00%)		2 (13.33%)		1 (6.67%)		
Stage						*p* = 0.169
I (n = 4) (13.33%)		3 (20.00%)		1 (6.67%)		
II (n = 6) (20.00%)		5 (33.33%)		1 (6.67%)		
III (n = 10) (33.33%)		3 (20.00%)		7 (46.67%)		
IV (n = 10) (33.33%)		4 (26.67%)		6 (40.00%)		
Histology						*p* = 0.591
Squamous Cell Carcinoma (n = 26) (86.67%)	Others (n = 4) (13.33%)	SCC	Others	SCC	Others	
		12 80.00%	3 20.00%	14 93.33%	1 6.67%	
Differentiation						*p* = 0.693
Well differentiated (n = 4) (13.79%)		1 (7.14%)		3 (20.00%)		
Moderately differentiated (n = 22) (75.86%)		11 (78.57%)		11 (73.33%)		
Poorly differentiated (n = 3) (10.34%)		2 (14.29%)		1 (6.67%)		

**Table 3:** Treatment characteristics of head and neck cancer patients.

Treatment Characteristics		Groups				*p* Value
		ONS (n = 15)		No ONS (n = 15)		
Surgery						*p* = 0.256
No	Yes	No	Yes	No	Yes	
(n = 19) (63.33%)	(n = 11) (36.67%)	11 73.33%	4 26.67%	8 53.33%	7 46.67%	
Concurrent chemoradiotherapy						*p* = 0.215
No	Yes	No	Yes	No	Yes	
(n = 22) (73.33%)	(n = 8) (26.67%)	13 86.67%	2 13.33%	9 60.00%	6 40.00%	
Radiotherapy only						*p* = 0.121
No	Yes	No	Yes	No	Yes	
(n = 10) (33.33%)	(n = 20) (66.67%)	3 20.00%	12 80.00%	7 46.67%	8 53.33%	
Induction chemotherapy and radiotherapy						*p* = 0.591
No	Yes	No	Yes	No	Yes	
(n = 26) (86.67%)	(n = 4) (13.33%)	14 93.33%	1 6.67%	12 80.00%	3 20.00%	
Treatment completion						*p* = 1.000
No	Yes	No	Yes	No	Yes	
(n = 2) (6.67%)	(n = 28) (93.33%)	1 6.67%	14 93.33%	1 6.67%	14 93.33%	
Radiotherapy total dose		ONS (n = 14)		No ONS (n = 14)		*p* = 0.002
70 Gy (n = 10) (35.71%)		1 (7.14%)		9 (64.29%)		
66 Gy (n = 4) (14.29%)		4 (28.57%)		0 (0.00%)		
65.25 Gy (n = 1) (3.67%)		1 (7.14%)		0 (0.00%)		
63 Gy (n = 4) (13.33%)		2 (14.29%)		0 (0.00%)		
60 Gy (n = 11) (39.29%)		6 (42.86%)		5 (35.71%)		
Chemotherapy regime		ONS (n = 15)		No ONS (n = 15)		*p* = 0.205
No chemotherapy (n = 20) (66.67%)		12 (80.00%)		8 (53.33%)		
Cisplatin (n = 6) (20.00%)		2 (13.33%)		4 (26.67%)		
TPF and Cisplatin (n = 2) (6.67%)		0 (0.00%)		2 (13.33%)		
Cisplatin and 5FU (n = 1) (3.33%)		0 (0.00%)		1 (6.67%)		
Cisplatin and Gemcitabine (n = 1) (3.33%)		1 (6.67%)		0 (0.00%)		
Number of Oral Nutritional Supplement weeks		ONS (n = 15)				
Less than 4 weeks (n = 4) (13.33%)		4 (26.67%)				
4 or more weeks (n = 11) (36.67%)		11 (73.33%)				

**Table 4:** Comparison of weight (kg) between the two studied groups.

Body Weight (kg)	Groups		*p* Value
	ONS (n = 15)	No ONS (n = 15)	
Before start of radiotherapy	77.80 (63.50–102.10)	75.70 (56.30–96.60)	*p* = 0.373
At the middle of radiotherapy course	76.10 (64.10–104.50)	74.85 (55.60–93.70)	*p* = 0.421
At the end of radiotherapy course	73.90 (64.30–103.30)	72.95 (55.00–90.10)	*p* = 0.383
Percentage change (%) (middle vs. before start of radiotherapy)	−2.32 (−3.34–−2.35)	−1.81 (−5.39–−1.63)	*p* = 0.581
Percentage change (%) (end vs. before start of radiotherapy)	−2.12 (−8.11–−2.50)	−5.55 (−9.49–−2.31)	*p* = 0.009 *
Percentage change (%) (end vs. middle of radiotherapy course)	−1.02 (−6.00–−1.57)	−3.24 (−6.97–−1.08)	*p* = 0.001 *

* statistically significant.

**Table 5:** Comparison of body mass index (kg/m^2^) between the two studied groups.

Body Mass Index (kg/m^2^)	Groups		*p* Value
	ONS (n = 15)	No ONS (n = 15)	
Before start of radiotherapy	28.20 (21.72–29.83)	28.15 (21.00–29.30)	*p* = 0.254
At the middle of radiotherapy course	28.20 (21.92–30.53)	27.37 (20.24–28.79)	*p* = 0.215
At the end of radiotherapy course	27.64 (21.99–30.18)	26.34 (19.53–27.97)	*p* = 0.054
Percentage change (%) (middle vs. before start of radiotherapy)	−2.32 (−3.34–−2.35)	−1.81 (−5.39–−1.63)	*p* = 0.581
Percentage change (%) (end vs. before start of radiotherapy)	−2.12 (−8.11–−2.50)	−5.55 (−9.49–−2.31)	*p* = 0.009 *
Percentage change (%) (end vs. middle of radiotherapy course)	−1.02 (−6.00–−1.57)	−3.24 (−6.97–−1.08)	*p* = 0.001 *

* statistically significant.

**Table 6:** Comparison of body fat mass (kg) between the two studied groups.

Body Fat Mass (kg)	Groups		*p* Value
	ONS (n = 15)	No ONS (n = 15)	
Before start of radiotherapy	24.50 (14.30–45.10)	22.70 (10.30–26.40)	*p* = 0.071
At the middle of radiotherapy course	23.85 (13.60–45.20)	20.40 (10.10–27.80)	*p* = 0.168
At the end of radiotherapy course	23.45 (13.20–45.50)	19.30 (10.00–26.60)	*p* = 0.073
Percentage change (%) (middle vs. before start of radiotherapy)	−5.58 (−11.84–−5.03)	−2.18 (−13.82–−6.04)	*p* = 0.520
Percentage change (%) (end vs. before start of radiotherapy)	−6.98 (−13.13–−11.32)	−5.91 (−16.74–−1.23)	*p* = 0.183
Percentage change (%) (end vs. middle of radiotherapy course)	−0.51 (−6.95–−8.45)	−4.87 (−10.45–−0.99)	*p* = 0.001 *

* statistically significant.

**Table 7:** Comparison of fat-free mass (kg) between the two studied groups.

Fat-Free Mass (kg)	Groups		*p* Value
	ONS (n = 15)	No ONS (n = 15)	
Before start of radiotherapy	51.55 (41.10–63.70)	55.50 (43.90–72.30)	*p* = 0.556
At the middle of radiotherapy course	53.10 (40.90–65.50)	54.00 (43.30–68.10)	*p* = 0.727
At the end of radiotherapy course	52.50 (38.80–62.40)	53.60 (43.30–66.70)	*p* = 0.872
Percentage change (%) (middle vs. before start of radiotherapy)	−0.34 (−3.17–−4.39)	−1.24 (−5.81–−2.87)	*p* = 0.118
Percentage change (%) (end vs. before start of radiotherapy)	−2.13 (−5.60–−2.81)	−3.65 (−7.75–−1.15)	*p* = 0.012 *
Percentage change (%) (end vs. middle of radiotherapy course)	−0.43 (−5.13–−3.48)	−2.54 (−6.24–−0.73)	*p* = 0.098

* statistically significant.

**Table 8:** Comparison of bone mass (kg) between the two studied groups.

Bone Mass (kg)	Groups		*p* Value
	ONS (n = 15)	No ONS (n = 15)	
Before start of radiotherapy	3.10 (2.50–4.30)	3.00 (2.50–3.70)	*p* = 0.188
At the middle of radiotherapy course	3.10 (2.60–4.30)	2.95 (2.60–3.50)	*p* = 0.310
At the end of radiotherapy course	3.00 (2.60–4.20)	2.95 (2.60–3.50)	*p* = 0.200
Percentage change (%) (middle vs. before start of radiotherapy)	0.00 (−3.70–−5.00)	0.00 (−6.45–−3.70)	*p* = 0.188
Percentage change (%) (end vs. before start of radiotherapy)	0.00 (−13.95–−5.00)	−3.00 (−9.68–−3.70)	*p* = 0.099
Percentage change (%) (end vs. middle of radiotherapy course)	0.00 (−5.13–−3.48)	0.00 (−6.25–0.00)	*p* = 0.486

**Table 9:** Comparison of radiotherapy side effects between the two studied groups.

Radiotherapy side effects		Groups				*p* Value
		ONS (n = 15)		No ONS (n = 15)		
Nausea		n = 14		n = 14		*p* = 0.589
NO	YES	NO	YES	NO	YES	
(n = 5) (17.86%)	(n = 23) (82.14%)	2 14.29%	12 85.71%	13 92.86%	1 7.14%	
Dysphagia		n = 14		n = 14		*p* = 1.000
NO	YES	NO	YES	NO	YES	
(n = 24) (85.71%)	(n = 4) (14.29%)	11 78.57%	3 21.43%	3 21.43%	11 78.57%	
Food intake		n = 14		n = 14		*p* = 0.058
Solid	Liquid	Solid	Liquid	Solid	Liquid	
(n = 15) (53.57%)	(n = 13) (46.43%)	10 71.43%	4 28.57%	5 35.71%	9 64.29%	
Dermatitis		n = 14		n = 14		*p* = 0.463
NO	Grade 1	NO	Grade 1	NO	Grade 1	
(n = 2) (7.14%)	(n = 26) (92.86%)	2 14.29%	12 85.71%	0 0.00%	14 100.00%	
Mucositis Grade		n = 14		n = 14		*p* = 0.396
No (n = 1) (3.57%)		1 (7.14%)		0 (0.00%)		
Grade 1 (n = 21) (75.00%)		11 (78.57%)		10 (71.43%)		
Grade 2 (n = 3) (10.71%)		2 (14.29%)		1 (7.14%)		
Grade 3 (n = 3) (10.71%)		0 (0.00%)		3 (21.43%)		
Xerostomia		n = 14		n = 14		*p* = 0.256
No (n = 1) (3.57%)		1 (7.14%)		0 (0.00%)		
Mild (n = 15) (53.57%)		9 (64.29%)		6 (42.86%)		
Moderate (n = 12) (42.86%)		4 (28.57%)		8 (57.14%)		

## Data Availability

The datasets used and analyzed during the current study are available from the first author on reasonable request.
